# An ecological approach to measuring synchronization abilities across the animal kingdom

**DOI:** 10.1098/rstb.2020.0336

**Published:** 2021-10-11

**Authors:** Molly J. Henry, Peter F. Cook, Koen de Reus, Vivek Nityananda, Andrew A. Rouse, Sonja A. Kotz

**Affiliations:** ^1^ Research Group ‘Neural and Environmental Rhythms', Max Planck Institute for Empirical Aesthetics, Grüneburgweg 14, 60322 Frankfurt am Main, Germany; ^2^ Department of Psychology, New College of Florida, 5800 Bayshore Rd, Sarasota, FL 34234, USA; ^3^ Comparative Bioacoustics Group, Max Planck Institute for Psycholinguistics, Wundtlaan 1, 6525 XD Nijmegen, The Netherlands; ^4^ Artificial Intelligence Lab, Vrije Universiteit Brussel, Boulevard de la Plaine 9, 1050 Ixelles, Belgium; ^5^ Biosciences Institute, Newcastle University, Newcastle Upon Tyne, NE2 4HH, UK; ^6^ Department of Psychology, Tufts University, 419 Boston Ave, Medford, MA 02155, USA; ^7^ Department of Neuropsychology and Psychopharmacology, Faculty of Psychology and Neuroscience, Maastricht University, Universiteitssingel 40, 6200 MD Maastricht, The Netherlands

**Keywords:** synchronization, timing, flexibility, motivation, ecological design

## Abstract

In this perspective paper, we focus on the study of synchronization abilities across the animal kingdom. We propose an ecological approach to studying nonhuman animal synchronization that begins from observations about when, how and why an animal might synchronize spontaneously with natural environmental rhythms. We discuss what we consider to be the most important, but thus far largely understudied, temporal, physical, perceptual and motivational constraints that must be taken into account when designing experiments to test synchronization in nonhuman animals. First and foremost, different species are likely to be sensitive to and therefore capable of synchronizing at different timescales. We also argue that it is fruitful to consider the latent flexibility of animal synchronization. Finally, we discuss the importance of an animal's motivational state for showcasing synchronization abilities. We demonstrate that the likelihood that an animal can successfully synchronize with an environmental rhythm is context-dependent and suggest that the list of species capable of synchronization is likely to grow when tested with ecologically honest, species-tuned experiments.

This article is part of the theme issue ‘Synchrony and rhythm interaction: from the brain to behavioural ecology’.

## 1. Introduction

Humans synchronize flexibly with each other and with environmental rhythms, and enjoy doing so: we share music and dance together, and bounce our babies. These synchronized behaviours contribute to social bonding and group coherence [1,2]. Many nonhuman animal species also engage in synchronized displays. Hundreds of fireflies in Southeast Asia flash in unison [3], creating a ‘beaconing’ effect to attract mates [4]. Groups of up to approximately 10 orthopterans and frogs coordinate their calls [5–8], creating choruses that are louder than any single organism signalling alone [9], improving the probability of reproductive success and serving an anti-predatory function [10,11]. Synchronization between conspecifics has also been observed in bioluminescent fish and marine crustaceans [12,13] as well as claw-waving crabs [14]. Despite its ubiquity across the animal kingdom, *rhythmic synchronization* is accomplished via different mechanisms, serves different functions and is observed in different contexts for different species.

We propose an ecologically honest approach to studying synchronization abilities in nonhuman animals in the milliseconds-to-seconds range; we are not concerned with synchronization on circadian or seasonal timescales. Our approach involves mapping the possibility space that describes when and why synchrony might be possible in the wild, given the temporal, sensory, motor and motivational constraints on a species' natural repertoire. Taken together with a survey of the latent flexibility of an animal's synchronization behaviours, these considerations will inform us about the range of natural and in-laboratory conditions in which we can experimentally test the capabilities and limitations of animal synchronization, respecting the full range of potential forms synchronization can take on.

We must first consider what it means for any animal (human or nonhuman) to be capable of synchronization. Nonlinear dynamics prescribes a set of preconditions for synchronization, from which we derive behavioural and theoretical consequences [15]. Notably, this definition of synchronization is a general one and can be applied as easily to a pendulum as it can to animal synchronization.

First, *the synchronizer must generate its own rhythm*. For any animal to be capable of synchronization, it must first be capable of auto-generating a rhythmic behaviour that can then become synchronized to an environmental rhythm. Critically, this distinguishes synchronization from repetitive reactions to repetitive stimuli, which may occur independent of the presence of regular rhythmic structure in a stimulus. Although these two means to temporal coordination are not necessarily separable based on data analysis alone, one way to empirically distinguish between synchronization and repetitive reaction is to focus on *anticipation* of the stimulus by the response [16]. Human finger taps occur slightly before stimulus events, a phenomenon termed *negative mean asynchrony* [17]. By contrast, macaque monkeys' movements lag behind repetitive stimulus events [18], though this time lag is shorter than standard reaction times, ruling out a completely reactive strategy. In interactions between katydids of the genus *Mecopoda*, one male often consistently leads another [19,20], though this phenomenon might simply reflect one male being able to call faster than the other. Nonetheless, independent of the precise underlying mechanism, many species demonstrate signatures of anticipatory synchronization behaviour that are distinguishable from serial reactions to repetitive stimulation.

Second, *a synchronizer adjusts its own rhythm during interaction with an environmental rhythm*. That is, a synchronizer adopts a common frequency as, and therefore a fixed phase relationship with, a stimulus rhythm. Notably, a fixed phase relationship does not imply temporal coincidence, as anti-phase relationships are also possible between synchronized systems [15]; natural anti-phase synchronization is exhibited by, for example, harbour seals (*Phoca vitulina*: [21]), bottlenose dolphins (genus *Tursiops*: [22]), frogs (*Physalaemus pustulosus*: [23]) and katydids (*Ephippiger ephippiger*: [6]). Arguably, the most critical implication of this precondition is *tempo-flexibility* [24]: an organism lacking tempo-flexibility could only synchronize with an environmental rhythm at precisely its own intrinsic rate, and it is obvious that such a tempo-restricted form of synchronization would be of limited practicality.

Third, *adjustments of a synchronizer's rhythm occur in a limited range of mismatch with the environmental rhythm (detuning)*. Synchronization is accomplished most easily when the rate of the organism's own rhythm closely matches the rate of the stimulus rhythm. No organism possesses unlimited flexibility; thus, as the difference between the intrinsic and environmental rates increases, synchronization is less successful. The natural consequence of this precondition is a *restricted range of tempi* around one's own preferred rate with which an organism can accomplish 1 : 1 synchronization. When an environmental rhythm becomes too extreme, katydids switch to a 1 : 2 or 2 : 1 synchronization mode [25] or synchronize with unstable, constantly changing phase relationships [19].

Thus, empirically, synchronization can be identified based on the presence of anticipation and tempo-flexibility within a restricted range. This definition of synchronization is mechanistically, motivationally and cognitively agnostic. This is important, as there is no privileged mechanism for synchrony across the animal kingdom. In fact, the mechanisms supporting synchronization differ within orthopterans [6,19,20,26,27] and within fireflies [3,28], and humans can achieve synchrony by engaging different neural mechanisms depending on the disease state of an individual [29]. Both anticipation and tempo-flexibility have been recognized in the comparative literature as critical features of synchrony [16,24]. However, this is the first acknowledgement we are aware of that tempo-flexibility must necessarily be rate-restricted. This definition has clear and actionable consequences for empirical assessments of synchrony in different species that necessitate the adoption of an ecological approach: we must establish the natural range of behavioural periods species and individuals produce to fairly assess their synchronization capabilities in the laboratory.

We discuss what we consider to be the most important temporal, physical, sensory and motivational constraints that must be considered *together* when designing experiments to test synchronization abilities. First, we examine the range of timescales that different species act within and are likely to be sensitive to (§2). Second, we argue that it is fruitful to consider the latent flexibility of synchronization across a number of dimensions (§3). Finally, we discuss the importance of an animal's motivational state for demonstrating synchronization abilities (§4). We review findings from both field and laboratory studies as, for many species, only laboratory data are available. However, we argue that these studies underestimate synchronization abilities across the animal kingdom, and we lay out an alternative approach that starts from an animal's *natural* repertoire.

## 2. Time-scale constraints on synchronization

Most animals producing rhythmic behaviours do so across a range of possible rates that depends on context and motor system. For example, typical human gait ranges between 0.7 and 1.2 Hz (42 and 72 beats per minute, BPM) [30], while the mean movement frequency across all daily behaviours is closer to 2.0 Hz (120 BPM) [31]. Interestingly, it is this latter number that best matches human preferences for tempi in popular music [32]. Comparative work exploring nonhuman animal synchronization often bases stimulus design on these human rate preferences. However, to select appropriate stimulus rates for assessing synchrony, we must target tempi within an animal's natural range for any selected behaviour. What sets the limits of these ranges, and how might we chart them for different species?

**Figure 1.**
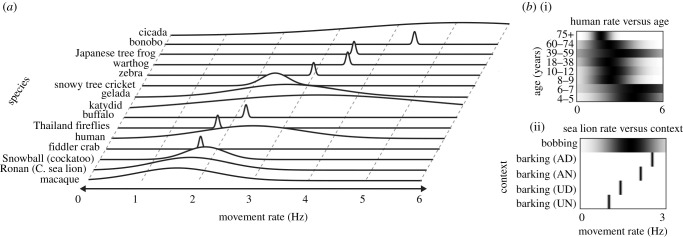
(*a*) A framework for mapping and considering rhythmic abilities across species. Line plots show preferred rates and empirical ranges for synchronization across a sampling of species from table 1. Note: This figure is intended to be illustrative of how our proposed ecological approach could work, rather than being definitive regarding the relevant rates for a particular species. With more comprehensive data, further markers could be included to delineate relevant contextual factors. For visualization, data are modelled as Gaussian functions with full-width-half-maximum (FWHM) equal to the empirically documented synchronization range, when a range was available in the literature. When this was not the case, and only a single value was available, FWHM was set equal to a default minimum value of 0.05 Hz (fiddler crab, Thailand fireflies, buffalo, zebra, warthog, Japanese tree frog, bonobo). (*b*) Preferred rates and rate limits (flexibility) are context-dependent). (i) Human preferred rates slow over the lifespan, and the range of spontaneously produced rates is likewise not stable over age, becoming especially restricted over age 75; age-range data estimated from Alexander & Moore [38]. (ii) Sea lion vocalizations (barks) are produced at different rates depending on the medium (air versus water) and social context (directed versus undirected); AD = air directed, AN = air non-directed, UD = underwater directed, UN = underwater non-directed. Bark rates are plotted underneath the range of rates at which Ronan successfully synchronizes head bobs. In both panels, grayscale plots represent Gaussian functions, with dark colours corresponding to peaks.

Skeletal and motor systems set inherent limits on the movement frequencies of animals. Movement rates may be ‘soft assembled’, that is, a byproduct of specific effector properties acting on and reacting to the environment. Thus, animal effector systems will naturally ‘prefer’ certain movement rates in certain contexts. An empirical assessment of stepping frequency in animals of different sizes has found that total body mass, effector size and jointing account for much of the variability across species and animals of different overall sizes ([33]; table 1). Such innate tendencies will vary with age (figure 1) and sex, both of which can affect joint elasticity and body size. Gait can be easily measured across species and may be a good starting point for establishing rate capabilities across species, but will not directly predict movement period across all motor systems and environments. For example, movement patterns may also vary between media: California sea lion vocalization (bark) rates underwater are slower than in air, possibly because of the increased energetic requirements of sound production underwater (table 1 and figure 1).

**Table 1.** Periodic behavioural rates across different species and contexts. Periodic movement rates were collected from a wide range of empirical and observational studies. The first column specifies the species and the source. The second column specifies the type of movement or behaviour. The third column lists the recorded behavioural rate or rates. These are presented as a range when available, otherwise as a single value. In certain cases, these numbers were estimated from graphical representations where numerical tables were not available. The fourth column addresses the context for measurement, whether the behaviour was elicited in some way by humans (e.g. the animal was chased to run, or trained to produce a behaviour) or occurred spontaneously as part of the animal's natural repertoire. In addition, laboratory studies are further labelled here as experimental. The fifth column addresses whether the behaviour is likely to be social or not. Behaviours are listed as yes (almost certainly produced in a naturally occurring socially relevant context), ‘no,’ (almost certainly not produced in a naturally occurring socially relevant context) or ‘maybe.’ Behaviours coded as ‘maybe’ are further specified as: ‘maybe(a)’—locomotion behaviour sometimes used in herd/flock contexts, ‘maybe(b)’—used sometimes in social contexts but the function of the behaviour is not agreed on and ‘maybe(c)’—trained or instructed behaviours that would not typically occur in natural contexts but are elicited in experimental settings that may have a social or parasocial context. Italicized rows correspond to the data visualized in figure 1.

species	behaviour	cycles/second (Hz)	context	social?
bobwhite [30]	gait	1.5–4.5	naturally occurring	maybe(a)
guineafowl [30]	gait	0.7–3	naturally occurring	maybe(a)
turkey [30]	gait	1.2–2.2	naturally occurring	maybe(a)
emu [30]	gait	1–1.5	naturally occurring	maybe(a)
rhea [30]	gait	0.8–1.5	naturally occurring	maybe(a)
ostrich [30]	gait	0.8–1.3	naturally occurring	maybe(a)
human [30]	gait	0.7–1.2	naturally occurring	maybe(a)
painted quail [30]	gait	2–7	naturally occurring	maybe(a)
macaque [34]	teeth chattering	5.7 mean	naturally occurring	yes
C. sea lion [35]	in air non-directed barking	2.1 mean	naturally occurring	yes
C. sea lion [35]	in air directed barking	2.5 mean	naturally occurring	yes
C. sea lion [35]	underwater non-directed barking	1	naturally occurring	yes
C. sea lion [35]	underwater directed barking	1.4	naturally occurring	yes
bonobo [36]	contest hooting	3–4	naturally occurring	yes
*Thailand fireflies* [3]	*flashing*	*1.8*	*naturally occurring*	*yes*
ostracode crustaceans [37]	flashing	0.3–2	naturally occurring	yes
ponyfish [12]	flashing	1.8	naturally occurring	yes
cicada [38]	ticking	16–25	naturally occurring	yes
*cicada* [38]	*buzzing*	*3.6–6.7*	*naturally occurring*	*yes*
*katydid* [39]	*chirps (stridulation)*	*1.8–5.0*	*naturally occurring*	*yes*
katydid [39]	forewing strid	14–250	naturally occurring	yes
*snowy tree cricket* [40]	*chirps (stridulation)*	*2.2–2.6*	*naturally occurring*	*yes*
*Japanese tree frog* [41]	*vocal chorusing*	*3.5*	*naturally occurring*	*yes*
*fiddler crab* [14]	*claw waving*	*1.7*	*naturally occurring*	*yes*
dolphin [42]	surfacing while travelling	0.003–0.009	naturally occurring	yes
dolphin [43]	breathing interval	0.01–0.2	naturally occurring	yes
right whale dolphin [44]	burst pulses	4–11.3	naturally occurring	maybe(b)
bottlenose dolphin [22]	whistles	1–6	naturally occurring	yes
*gelada* [45]	*synch calls*	*2–3.5*	*naturally occurring*	*yes*
male mouse [46]	ultrasonic vocalizations	1.3–5.4	naturally occurring	yes
bat [47]	echolocation	5–200	naturally occurring	maybe(b)
human [31]	daily movement	2 mean	naturally occuring	maybe(b)
human [48]	music tempo	2 mean	averaged from popular songs	maybe(b)
*Snowball (cockatoo)* [49]	*bobbing*	*1.7–2.2*	*untrained to music, experimental*	*maybe(c)*
*bonobo* [50]	*one-hand drumming*	*4.2–4.8*	*untrained, experimental*	*maybe(c)*
giraffe [33]	gait	1.3	chased to run, field	maybe(a)
*buffalo* [33]	*gait*	*2.2*	*chased to run, field*	*maybe(a)*
eland [33]	gait	2.7	chased to run, field	maybe(a)
*zebra* [33]	*gait*	*3*	*chased to run, field*	*maybe(a)*
wildebeest [33]	gait	2.9	chased to run, field	maybe(a)
hartebeest [33]	gait	2.3	chased to run, field	maybe(a)
topi [33]	gait	2.5	chased to run, field	maybe(a)
*warthog* [33]	*gait*	*3.5*	*chased to run, field*	*maybe(a)*
impala [33]	gait	2.3	chased to run, field	maybe(a)
Thomson's gazelle [33]	gait	3	chased to run, field	maybe(a)
budgerigars [51]	pecking	0.6–2.2	trained, experimental	maybe(b)
*macaque* [18]	*tapping*	*1–2.2*	*trained, experimental*	*maybe(c)*
chimpanzee [52]	tapping	2.2–2.8	trained, experimental	maybe(c)
*Ronan (C. sea lion)* [53]	*bobbing*	*1.2–2.3*	*trained, experimental*	*maybe(c)*
human age 4–5 [54]	tapping	2.4–5	experimental	maybe(c)
human age 6–7 [54]	tapping	2.5–5.7	experimental	maybe(c)
human age 8–9 [54]	tapping	1.4–3.1	experimental	maybe(c)
human age 10–12 [54]	tapping	1.4–3.3	experimental	maybe(c)
*human age 18–38* [54]	*tapping*	*1.1–2.9*	*experimental*	*maybe(c)*
human age 39–59 [54]	tapping	1.3–4.4	experimental	maybe(c)
human age 60–74 [54]	tapping	1.2–2.9	experimental	maybe(c)
human age 75+ [54]	tapping	1.2–2.5	experimental	maybe(c)

While effectors may move at a range of tempi, synchronization will also be limited by an animal's perception. How do we know what rates might be well-perceived by an animal? Perception of rate and regularity can be assessed in controlled laboratory settings [55], but we may also infer the perceptual importance of specific rates by analysing the meaningful rate-varying stimuli that an animal encounters in its natural environment. Wild sea lions produce reliably isochronous barks at a mean rate of 2.1 Hz (126 BPM) [35]. Ronan the California sea lion, one of the most accomplished nonhuman experimental synchronizers, has shown top performance synchronizing with stimuli slightly faster than 2 Hz (120 BPM) [53]. Although Ronan's trained head-bobbing behaviour is likely not naturally occurring, the overlap of the tempi of wild sea lion barks and Ronan's beat synchronization performance is notable. When important stimuli in an animal's environment have reliable rates, and changes in those rates signal important information, as is true for sea lion vocalizations (figure 1), it is a safe bet that the animal is perceptually sensitive to stimulus rates in that range.

A preliminary assessment of prior literature on movement rates across species (table 1) indicates a large variability of rates and ranges within and across species, many far removed from the typical human-preferred rates used in many comparative experiments. Naturally occurring rates in nonhuman animal behaviour should thus anchor empirical assessment of animal synchronization. Animal bodies, perceptual systems and the behavioural ecology they support are not incidental to the question of animal rhythms, but rather absolutely central.

## 3. Latent flexibility of animal synchronization

### (a) Over what range of timescales might synchronization be observed?

Even the most flexible synchronizers we know of—humans—are constrained [56–59], with many tempi out of our range. Humans cannot synchronize with auditory stimuli at rates faster than approximately 7–10 Hz (420–600 BPM) [58,60,61] or slower than approximately 0.5 Hz (30 BPM) [58]. This range shrinks for (static) visual stimuli, with which we fail to synchronize at rates faster than approximately 2.5 Hz (150 BPM) [61]. Nonhuman animals similarly demonstrate flexibility around their preferred rates (figure 1 and table 1). Macaque monkeys synchronize hand movements or saccades successfully within the range of approximately 1–2.2 Hz (60–132 BPM) [18,62,63], and budgerigars synchronize key pecks within the tested range of approximately 0.6–2.2 Hz (36–132 BPM) [51]. A bonobo drumming together with an experimenter synchronized in bouts within a narrow range of relatively fast rates between 4.2 and 4.8 Hz (252 and 288 BPM) [50]. Snowball, a sulfur-crested cockatoo, demonstrated bouts of synchrony at rates spanning approximately 1.7 and 2.2 Hz, but not outside this range [49]. Finally, Ronan showed anticipatory synchronization with rates in the window of approximately 1.2–2.4 Hz (72–144 BPM), but not faster [53,64]. *Mecopooda elongata*, a katydid species that chirps at a rate of approximately 0.5 Hz (30 BPM), synchronizes with external rhythms with periods as fast as 0.6 Hz (36 BPM), but not faster [19]. Treefrogs (*Eleutherodactylus coqui*), who call at rates around 0.4 Hz (24 BPM), demonstrate 1 : 1 phase locking for rates as fast as 0.6 Hz (36 BPM) [65], but switch to 1 : 2 phase locking to every second beat at faster intervals. Thus, all species in which synchronized behaviour has been observed demonstrate some degree of tempo-flexibility. However, the degree of flexibility varies across species and is likely an important clue to how easily a species demonstrates sensorimotor synchronization in the laboratory.

### (b) With which behavioural outputs might a species synchronize?

Humans can synchronize an array of behaviours to external (auditory) stimuli. Often, participants finger tap in time with a stimulus [58,59]. However, humans can perform the same task by drumming with a stick [66], tapping their toes [67], walking [68,69], dancing [70] or speaking [71,72]. Thus, humans are motorically flexible, maybe uniquely so [73–75]; but not all behavioural outputs are equally easy to synchronize to a stimulus. One hurdle to testing nonhuman animal synchronization in the laboratory is knowing *a priori* what behavioural output with which to ‘ask’ an animal to synchronize. For example, macaques can take years (up to 25 months; [18]) to learn to synchronize hand movements with a stimulus rhythm; then, once they have reached ‘criterion’ performance, their movements continue to lag behind the stimulus. Thus, an animal might fail at an in-laboratory synchronization task because we reinforce a suboptimal behavioural output, when another behavioural output, saccades in the case of macaques [76], might better showcase the flexibility they are capable of. Moreover, we cannot straightforwardly apply this lesson directly to other related species, as chimpanzees and bonobos for example spontaneously synchronize hand movements with rhythmic stimuli without any training [50,52,77]. We argue that we will better identify candidates for suitable behavioural outputs if we focus on the spontaneous behaviours of nonhuman animals in their own natural environments, for example, the timing of steps in horses or the swinging of arms in primates or trunks in elephants.

### (c) Can synchronization be accomplished across multiple modalities?

Humans can synchronize motor outputs with auditory [59], visual [78] and vibrotactile stimulus rhythms [79]. Synchronization is best when stimuli are auditory. Static visual rhythms elicit the poorest synchronization performance, which can critically be improved by adding spatial information to visual rhythms [80–85]. This is important, because an animal may appear to be incapable of synchronizing with an inappropriate stimulus modality, or when experimenters have failed to capture a critical dimension in stimulus design. Consider flocking or swarming animals (see §4), who might synchronize with complex optic flow information for which we might not even fully know how to design appropriate stimuli so that we could test their capabilities [86].

Human synchronization abilities are proposed to be unique in that the behavioural response (clapping, singing, etc.) occurs in a different modality than the stimulus [16]. That is, humans tap their fingers or feet to a sound, while fireflies synchronize their flashes with other flashes. Synchronization of ‘like with like’ is proposed to be a more rigid form of synchronization than that of humans, Snowball and Ronan. However, this criticism sells short the complexity of sensorimotor synchronization in like-with-like synchronizers while simultaneously overselling the complexity of cross-modal synchrony. In katydids, the acoustic stimulus is processed by the auditory nervous system, but the acoustic response is produced in the motor system by a neural oscillator that drives the striking together of specialized structures on the katydids' wings [25,87,88]. Thus, any kind of synchronized animal behaviour is *sensorimotor* synchronization, even if the response is in the same modality as the stimulus.

## 4. The importance of motivational state for testing synchronization abilities

Whether in the wild or laboratory, animals will not act without motivation. It is self-evident but often overlooked when reporting negative results that an animal may fail to perform due to lack of capability *or* willingness. An animal's motivational state is modulated by intrinsic and extrinsic factors and the interaction between them. Intrinsic factors relate to the satisfaction of physiological needs, and extrinsic factors to stimuli present in an animal's environment [89]. The relative importance of these factors will differ based on context, for example, whether an animal is captive or wild. We provide specific examples from three types of overlapping and by no means comprehensive motivational contexts: motivation in mating, locomotive and social-coordination contexts. We illustrate each context with examples from the animal kingdom and demonstrate the importance of considering motivational states in designing species- and context-specific behavioural studies. Importantly, our use of the term motivation is intended to capture the drive to act in a particular way without assuming an animal's recognition of the reason to do so.

### (a) Motivation in mating contexts

Synchrony is often observed in collective mating-display behaviours that have evolved due to selective pressures on mating and reproduction [10,90]. In fiddler crabs, males form small groups and wave their enlarged major claws up and down in near-perfect synchrony [91]. In several species of katydids and grasshoppers, males signal in synchrony with their neighbours [10]. However, synchronous displays arise out of different motivations, although on the surface they might appear very similar. For example, behavioural displays characterized by *adaptive synchrony*, which serves a cooperative function, attract a higher number of females [9,26,92,93] while simultaneously helping individual species members avoid detection by predators [11]. In other species, however, including katydids, *incidental synchrony* arises as a consequence of males competing with one another to emit the leading signal [87,88], which is preferred by females [91,94]. Motivations in mating contexts are unlikely to be equally strong at all times and may only be present in a mating season, limiting the available time window for observing mating-motivated rhythmic behaviours as well as our ability to test animals outside of these conditions.

### (b) Motivation in locomotion contexts

Most animals move around within their natural environments to use resources and avoid predators. In doing so, bipedal and quadrupedal animals move with symmetrical gaits, a natural source of rhythmic behaviour (see §2; table 1). However, rhythmic locomotive behaviour is not limited to gaits. For example, head-bobbing is a stabilizing reflex and helps facilitate walking in some birds, such as quails, by synchronizing head movements with the pitch of the trunk [95]. Animals in locomotive contexts may also move in groups, and remarkable temporal coordination has been observed in bird flocks, fish schools and dolphin pods. Coordinated group movement appears to provide an energetic advantage relative to solo locomotion [96,97]. Flying in formation may provide birds with an aerodynamic advantage relative to solo flight, but has also been proposed to serve a social function [96]. Swimming in schools serves an anti-predatory purpose [98], and improves efficient foraging activity [97]. In rough-toothed dolphins (*Steno bredanensis*), synchronized swimming is thought to be an energetic travelling adaptation that also facilitates eavesdropping [99]. In long-finned pilot whales, coordinated swimming provides an anti-predatory benefit [100] and shows affiliation, demonstrating that a particular behaviour may simultaneously belong to different motivational categories.

Designing experiments to test synchronization of locomotive behaviours must consider the necessary space an animal needs to execute these behaviours, especially in laboratory conditions. For example, dolphins in captivity do not have large pools to swim in, so their natural behavioural repertoire in captivity will be limited relative to natural conditions. Moreover, many individual behaviours only become apparent when the individual becomes part of a group. Thus, studying locomotive behaviours of flocking and schooling species on the individual level may miss complex capacities for synchronization.

### (c) Motivation in social-coordination contexts

There are many benefits to rhythmic social interaction. Interpersonal synchrony between human adults increases affiliation [101], and children that make music together engage in more prosocial behaviour [102]. In the wild, bonobos and some birds engage in asynchronous calling to avoid overlapping their calls with conspecifics with which they have close social ties [103]. The degree of signal coordination in bird duets represents coalition quality [104], and behavioural coordination in zebra finches is enhanced by familiarity [105]. Many species seem capable of adapting their call timing relative to that of a conspecific and are motivated to do so to increase efficient communication or to strengthen social bonds. A socially motivated rhythmic behaviour may not be elicited in a nonsocial context. Thus, in addition to charting the range of rates produced by animals in their natural environment, we may also benefit from assessing the context in which the behaviour occurs (table 1 and figure 1).

## 5. Conclusion and future directions

We have introduced an ecologically honest approach to studying nonhuman animal synchronization abilities and attempted to illustrate how this approach can be applied across the animal kingdom by surveying the temporal, sensory, motor and motivational constraints that influence naturally produced rhythmic behaviour. Although by no means exhaustive, we provide representative examples from species belonging to a number of different animal clades. Some of these species are known to display synchronized behaviour, and some, to our knowledge, have not been considered in the context of studying synchronization (table 1). Our goal was to demonstrate that animal rhythmic behaviour is context-dependent. We reviewed data from fieldwork, laboratory studies and hybrid designs. For many species, the only existing data points come from the laboratory. We suggest that the relative dearth of data on natural rhythmic behaviour for many species simultaneously handicaps our assessments of synchronization abilities and demands the ecological approach we propose here. We show that ecologically valid experiments studying rhythmic behaviours will benefit from species-specific designs [106] that consider the temporal niche, frequency of occurrence and function of natural behaviours. The ecological approach is a broadly encompassing method to study synchronization, which can and should be applied to humans and nonhumans. It bears emphasis that our admittedly cursory consideration of the comparative data on rate ranges, flexibility in rhythm generation and motivational context-dependence yields a striking degree of multidimensional variability between species, well beyond what has been carefully assessed in comparative laboratory experiments.

Our approach necessitates a close interplay between experimental and fieldwork and is thus by its very nature interdisciplinary. Generating a map of the behavioural possibility space for any species necessitates fieldwork to first identify why, when and how members of that species produce rhythmic and/or synchronized behaviours in their natural environments. In turn, we gain the capacity to design well-informed species-specific experiments to test synchronization abilities. Work on katydids (*Mecopoda* 'Chirper', *Neoconocephalus spiza*, *Ephippiger ephippiger*), grasshoppers (*Ligurotettix planum*), fireflies (*Pteroptyx malaccae*, *Pteroptyx valida*) and frogs (*Hyla cinerea*, *Pseudacris streckceri*, *Hyperolius marmoratus*) can be considered a gold standard. Fieldwork might be buttressed by sophisticated computational approaches, which are becoming more accessible and more user friendly. In particular, algorithmic approaches [107] might help us to identify the timescales, contexts and effectors that characterize the production of rhythmic behaviours, even when those rhythms exist outside of humans' temporal niche. Moreover, a broader experimental approach will integrate converging evidence from traditional sensorimotor synchronization experiments with *implicit* paradigms where synchronization is spontaneous and so does not need to be trained [77] and *neuroscientific* paradigms where the neural machinery that supports synchronization abilities or their precursors might be better understood [108].

To conclude, constraining our search for experimental parameters based on wild behaviour and ecologically relevant factors provides a meaningful approach to gaining a more complete understanding of which animal species are capable of synchrony.

## Data accessibility

This article has no additional data.
